# Supplementary material for: simCAS: an embedding-based method for simulating single-cell chromatin accessibility sequencing data
Source: Bioinformatics. 2023 Jul 26;39(8):btad453. doi: 10.1093/bioinformatics/btad453 (PMC10394124; doi:10.1093/bioinformatics/btad453)
Supplement: btad453_Supplementary_Data [file btad453_supplementary_data.pdf]

Supplementary Information for

simCAS: an embedding-based method for simulating single-cell Chromatin Accessibility Sequencing data

Chen Li[1,#], Xiaoyang Chen[1,#], Shengquan Chen[2], Rui Jiang[1,*], and Xuegong Zhang[1,*]

[1] Ministry of Education Key Laboratory of Bioinformatics, Bioinformatics Division at the Beijing National Research Center for Information Science and Technology, Center for Synthetic and Systems Biology, Department of Automation, Tsinghua University, Beijing 100084, China

[2] School of Mathematical Sciences and LPMC, Nankai University, Tianjin 300071, China

[*] Corresponding author: zhangxg@tsinghua.edu.cn, ruijiang@tsinghua.edu.cn

[#] These authors contributed equally: Chen Li, Xiaoyang Chen

Contents

Supplementary Notes **3**

Supplementary Figures **15**

Supplementary Tables **26**

References **27**

Supplementary Notes

Supplementary Note 1: Adapted simCAS framework with a Bernoulli assumption

Adapted simCAS with a Bernoulli assumption (referred to as simCAS_Bernoulli) is different from the original simCAS (referred to as simCAS_Poisson) in the following steps: estimation for distributions of statistics, parameter matrix correction and synthetic peak-by-cell matrix generation.

In the step of estimation for distributions of statistics, simCAS_Bernoulli only estimates distributions of library size (the number of aligned reads per cell) and peak summation (the sum of aligned reads per peak), except for cell non-zero proportion (the proportion of non-zero values per cell), which is repeated with library size under a Bernoulli assumption. The estimation of library size and peak mean of simCAS_Bernoulli is consistent with simCAS_Poisson. The estimated distributions of log-transformed library size and peak summation as $\mho_l$ and $\mho_p$ as in simCAS_Poisson.

In the step of parameter matrix correction, simCAS_Bernoulli performs the corrections by $\mho_l$ and $\mho_p$. For simplicity, here we used the symbols with the same meaning in simCAS_Poission. Suppose n_{cell}, n_{peak} and n'_{cell} denote number of simulated cells, number of simulated peaks and number of referenced cells in real data, respectively. After the low-dimensional embeddings generation and activation transformation as in simCAS_Poisson, we obtained the activated parameter matrix $\widehat{\boldsymbol{\Lambda}} \in \mathbb{R}^{n_{peak} \times n_{cell}}$. The library size correction is performed as follows:

(1) Multiply the CEV $\boldsymbol{l}$ and the CEM $\boldsymbol{C}$, and obtain $\hat{\boldsymbol{l}} \in \mathbb{R}^{1 \times n_{cell}}$. Each value of $\hat{\boldsymbol{l}}$ represents the weight of potential library size of each column vector (represents each cell) in $\widehat{\boldsymbol{\Lambda}}$.

(2) Randomly sample n_{cell} values from $\mho_l$ to form the synthetic library size set $L \in \mathbb{R}^{n_{cell}}$. Then assign the values of L to each column vector in $\widehat{\boldsymbol{\Lambda}}$ with the same order of the potential library size weights. Denote the value of L assigned to jth cell of $\widehat{\boldsymbol{\Lambda}}$ as l_j.

(3) For each column in $\widehat{\boldsymbol{\Lambda}}$ we perform a uniform correction. For the jth column vector $\hat{\boldsymbol{\lambda}}_{\cdot,j}$ in $\widehat{\boldsymbol{\Lambda}}$, sort it with an ascending order to get $\hat{\boldsymbol{\lambda}}^{sort}_{\cdot,j}$. Then search from the largest value to the lowest value of $\hat{\boldsymbol{\lambda}}^{sort}_{\cdot,j}$ to find an index I_j and a factor k_j to satisfy the equations:

$$\begin{cases} (\Sigma_{i=1}^{I_j} \hat{\lambda}^{sort}_{i,j} k_j) + (n_{peak} - I_j) = l_j \\ \hat{\lambda}^{sort}_{I_j,j} k_j < 1 \\ \hat{\lambda}^{sort}_{I_j+1,j} k_j \geq 1 \end{cases},$$

where $\hat{\lambda}_{i,j}^{sort}$ is the ith value of $\hat{\boldsymbol{\lambda}}_{\cdot,j}^{sort}$.

(4) Then multiply $\hat{\boldsymbol{\lambda}}_{\cdot,j}$ with k_j and set the values exceeding 1 to 1 to get the corrected parameters.

(5) Conduct the uniform correction for each column in $\widehat{\boldsymbol{\Lambda}}$ and acquire the library size corrected parameter matrix $\check{\boldsymbol{\Lambda}}$.

The peak mean correction is conducted with a similar operation:

(1) Randomly sample n_{peak} values from the $\mho_p$ to form the synthetic peak summation set $P \in \mathbb{R}^{n_{peak}}$. Then assign the values of P to each row vector (represents each peak) in $\check{\boldsymbol{\Lambda}}$ with the same order of the peak-wise summations in $\widehat{\boldsymbol{\Lambda}}$. Denote the value of P assigned to ith peak of $\widehat{\boldsymbol{\Lambda}}$ as p_j

(2) For each row in $\check{\boldsymbol{\Lambda}}$ we perform a uniform correction. For the ith row vector $\check{\boldsymbol{\lambda}}_{i,\cdot}$ in $\check{\boldsymbol{\Lambda}}$, sort it with an ascending order to get $\check{\boldsymbol{\lambda}}_{i,\cdot}^{sort}$. Then search from the largest value to the lowest value of $\check{\boldsymbol{\lambda}}_{i,\cdot}^{sort}$ to find an index I_i and a factor k_i to satisfy the equations:

$$\begin{cases} \left(\sum_{j=1}^{I_i} \check{\lambda}_{i,j}^{sort} k_i\right) + (n_{cell} - I_i) = n_{cell} \dfrac{p_j}{n'_{cell}} \\ \check{\lambda}_{i,I_i}^{sort} k_i < 1 \\ \check{\lambda}_{i,I_i+1}^{sort} k_i \geq 1 \end{cases},$$

where $\check{\lambda}_{i,j}^{sort}$ is the jth value of $\check{\boldsymbol{\lambda}}_{i,\cdot}^{sort}$.

(3) Then multiply $\check{\boldsymbol{\lambda}}_{i,\cdot}$ with k_i and set the values exceeding 1 to 1 to get the corrected parameters.

(4) Conduct the uniform correction for each row in $\check{\boldsymbol{\Lambda}}$ and acquire the peak mean corrected parameter matrix $\boldsymbol{\Lambda}$.

In the step of synthetic peak-by-cell matrix generation, we replaced the Poisson distribution with the Bernoulli distribution, of which the probability parameter is the corresponding parameter in $\boldsymbol{\Lambda}$.

Supplementary Note 2: Distributions for modeling peak summation

For simplicity of notation, we assume x as a random variable with no practical meaning in the following discussion. With specific modeling for the count of zero and one, the probability mass function of the Log-variant distribution is:

$$
\begin{aligned}
&f_{\text{Log-vairant}}(x; p, \pi_0, \pi_1) \\
&= \pi_0\delta_0(x) + \pi_1\delta_1(x) + (1 - \pi_0 - \pi_1) f_{Logarithmic}(x - 1; p) \\
&= \pi_0\delta_0(x) + \pi_1\delta_1(x) + (1 - \pi_0 - \pi_1)\frac{-1}{\ln(1-p)}\frac{p^{x-1}}{x-1},
\end{aligned}
$$

where $\delta_0(\cdot)$ and $\delta_1(\cdot)$ indicate the point mass function at zero and one, respectively, and π_0 and π_1 are the corresponding probabilities. p is a parameter ranging from 0 to 1 in Logarithmic distribution.

We also provide five discrete distributions for modeling peak summation as alternatives, namely Log-variantB (another variant of Logarithmic distribution different with Log-variant), NB (Negative Binomial), ZINB (Zero-Inflated Negative Binomial), NB-variant (a variant of Negative Binomial distribution) and ZIP distributions. For simplicity of notation, we assume x as a random variable with no practical meaning in the following discussion.

The probability mass function (PMF) of the Log-vairantB distribution is:

$$
\begin{aligned}
&f_{\text{Log-vairantB}}(x; p, \pi) \\
&= \pi_0\delta_0(x) + (1 - \pi_0) f_{\text{Logarithmic}}(x; p) \\
&= \pi_0\delta_0(x) + (1 - \pi_0)\frac{-1}{\ln(1-p)}\frac{p^{x}}{x}
\end{aligned}
$$

where $\delta_0(\cdot)$ indicates the point mass at zero, and π_0 is the corresponding probability. p is a parameter ranging from 0 to 1 in Logarithmic distribution.

The PMF of the NB distribution is:

$$
f_{\text{NB}}(x; r, p_0) = \frac{(x + r - 1)!}{(r - 1)!\, x!} p_0^x (1 - p_0)^x
$$

where r denotes the number of successes, and p_0 denotes the probability of success on each trial.

The PMF of the ZINB distribution is:

$$
f_{\text{ZINB}}(x; \pi_0, r, p_0) = \pi_0\delta_0(x) + (1 - \pi_0) f_{\text{NB}}(x; r, p_0)
$$

The PMF of the NB-variant distribution is:

$$
f_{\text{NB-variant}}(x; \pi_0, r, p_0) = \pi_0\delta_0(x) + (1 - \pi_0) f_{\text{NB}}(x - 1; r, p_0)
$$

The PMF of the ZIP distribution is:

$$f_{\text{ZIP}}(x; \pi_0, r, p_0) = \pi_0 \delta_0(x) + (1 - \pi_0) f_{\text{Poisson}}(x; \lambda) = \pi_0 \delta_0(x) + (1 - \pi_0) \frac{\lambda^x e^{-\lambda}}{x!}$$

where λ denotes the mean parameter of Poisson distribution.

Supplementary Note 3: Two operations for parameter matrix correction

First, we developed two activation functions adaptive to different modes. For the discrete or continuous mode, we provide a piecewise function $f_1(\cdot)$ by integrating an exponential function and a linear function as the activation function:

$$f_1(x) = \begin{cases} e^x, x \le k_1 \\ k_1 e^{k_1 - 1} x + e^{k_1} - k_1, x > k_1 \end{cases},$$

where k_1 is a parameter to control the variance of simulated data, and a higher k_1 value brings higher variance of peak accessibility. In this study k_1 is fixed to 2. For pseudo-cell-type mode, we expect the diversity within a cell type less than between different cell states, and a Sigmod-format function $f_2(\cdot)$ with a smaller slope is provided as the activation function:

$$f_2(x) = \frac{1}{1 + k_2^{-x}},$$

where k_2 is a parameter to adjust the steepness of the activation function curve and fixed to 2 in this study. After this operation, the parameter matrix $\widetilde{\boldsymbol{\Lambda}}$ is transformed into an activated parameter matrix $\widehat{\boldsymbol{\Lambda}}$.

Second, simCAS conducts correction with $\mho_l$, $\mho_c$ and $\mho_p$ in turn, to make simulated data preserve the cell-wise and peak-wise properties as with the real. For instance, simCAS performs the library size correction as follows:

(1) Multiply the CEV $\boldsymbol{l}$ and the CEM $\boldsymbol{C}$, and obtain $\hat{\boldsymbol{l}} \in \mathbb{R}^{1 \times n_{cell}}$.

(2) Randomly sample n_{cell} values from $\mho_l$ to form the synthetic library size set $L \in \mathbb{R}^{n_{cell}}$.

(3) Sort the elements of $\hat{\boldsymbol{l}}$ and values in L to obtain the sorting indices.

(4) Obtain the vector $\hat{\boldsymbol{l}}' \in \mathbb{R}^{1 \times n_{cell}}$ of the library sizes of synthetic cells by replacing the elements in $\hat{\boldsymbol{l}}$ with the sampled values from L one by one according to the sorting indices.

(5) For each column vector (represents each cell) in $\widehat{\boldsymbol{\Lambda}}$, divide each element by the sum of the vector, multiply all elements by the corresponding element (represents the associated cell) in $\hat{\boldsymbol{l}}'$, and obtain the corrected parameter matrix $\check{\boldsymbol{\Lambda}}$.

After correction of library size, simCAS obtains the corrected parameter matrix $\check{\boldsymbol{\Lambda}}$. simCAS then performs the peak summation correction as follows:

(1) Randomly sample n_{peak} values from $\mho_p$ to form the synthetic peak summation set $P \in \mathbb{R}^{n_{peak}}$.

(2) Sum the elements of each row vector in $\breve{\mathbf{\Lambda}}$, and obtain $\breve{\boldsymbol{p}} \in \mathbb{R}^{n_{peak} \times 1}$

(3) Sort the elements of $\breve{\boldsymbol{p}}$ and values in P to obtain the sorting indices.

(4) Obtain the vector $\breve{\boldsymbol{p}}' \in \mathbb{R}^{n_{peak} \times 1}$ of the peak summations of synthetic cells by replacing the elements in $\breve{\boldsymbol{p}}$ with the sampled values from P one by one according to the sorting indices.

(5) For each row vector (represents each peak) in $\breve{\mathbf{\Lambda}}$, divide each element by the sum of the vector, and then multiply all elements by the corresponding element (represents the associated cell) in $\breve{\boldsymbol{p}}'$. Then obtain the corrected parameter matrix $\acute{\mathbf{\Lambda}}$.

After correction of peak summation, simCAS obtains the corrected parameter matrix $\acute{\mathbf{\Lambda}}$. simCAS finally performs the cell sparsity correction as follows:

(1) Randomly sample n_{cell} values from $\mho_c$ to form the synthetic cell sparsity set $S \in \mathbb{R}^{n_{cell}}$.

(2) For the jth synthetic cell, randomly select a value in S without replacement, called s_j, as the sparsity of the cell.

(3) For the jth synthetic cell, obtain k_j and π_j by solving two nonlinear equations as follows:

$$\begin{cases} \sum_{m=1}^{n_{cell}} (1-\pi_j)(\acute{\lambda}_{m,j} k_j) = \sum_{m=1}^{n_{cell}} \acute{\lambda}_{m,j} \\ n_{peak}\pi_j + (1-\pi_j) \sum_{m=1}^{n_{cell}} e^{-\acute{\lambda}_{m,j} k_j} = n_{peak}(1-s_j) \end{cases}$$

where k_j and π_j denote a scaling factor and a zero-setting probability of the jth synthetic cell, respectively, and $\acute{\lambda}_{m,j}$ is the element in $\acute{\mathbf{\Lambda}}$ corresponding to the mth peak and the jth cell.

(4) For the jth synthetic cell, randomly set elements of the corresponding column vector to zero with the probability π_j, and then multiple the remaining non-zero elements by k_j.

(5) For each cell, performing the processing from (2) to (4), obtain the final parameter matrix $\mathbf{\Lambda}$.

Supplementary Note 4: Details of the optional steps in simCAS

simCAS could generates multi-batch data by incorporating the batch effects in the discrete simulation mode. For simulating data with the technical batch effect, we add the Gaussian noise on the corrected parameter matrix $\boldsymbol{\Lambda}$:

$$\boldsymbol{\Lambda}' = \boldsymbol{\Lambda} + \varepsilon_1 ,$$

where ε_1 is Gaussian noise sampled from $\mathcal{N}(\mu_t, \sigma_t^2)$. ε_1 can be deemed as technical variations when sequencing reads and results in an evident variation in library size values, and μ_t and σ_t control the variance of batches and degree of technical noise, respectively. $\boldsymbol{\Lambda}'$ and $\boldsymbol{\Lambda}$ generated from the common CEM guarantees the ground truth of same cells from different batches. With different levels of Gaussian noise added on $\boldsymbol{\Lambda}$, data with multiple technical batch effects can be generated. Note that the technical variance may be different in the cell types with different library sizes, we add Gaussian noise proportion to the average cell-wise summations of $\boldsymbol{\Lambda}$ in different cell populations.

The biological batch effect can be directly modeled by adding Gaussian noise to the PEM:

$$\boldsymbol{P}' = \boldsymbol{P} + \varepsilon_2 ,$$

where ε_2 is Gaussian noise sampled from $\mathcal{N}\left(\mu_b, \sigma_b^2\right)$. Then synthetic data with different batches is generated with a common CEM, which provides the ground truth of cells with same population.

The interactive peaks are constructed by assigning similar vectors in the PEM generation step. First, we pick up high chromatin accessibility regions to define hubs of interactive peaks, and then the correlation is added on the peaks of each hub. In a defined peak hub, randomly select some peaks as the interactive peaks and remaining peaks are non-interactive peaks. For interactive peaks in the hub, we first generate a general peak embedding vector $\widehat{\boldsymbol{p}} \in \mathrm{R}^{1\times n_{embed}}$, then the embedding vector of each interactive peak is generated by:

$$\boldsymbol{p}_{j'} = \widehat{\boldsymbol{p}} + \varepsilon_3 ,$$

where ε_3 is sampled from $\mathcal{N}(0, \sigma_p^2)$ and σ_p is a parameter to control the degree of co-accessibility among interactive peaks.

Supplementary Note 5: Details for baseline methods implementations, downstream analysis methods for benchmarking, and the procedure for data visualization

For simATAC simulation, we used simATAC (Navidi, et al., 2021) R package to directly simulate peak-by-cell matrix by regarding the peaks as bins in the input matrix. Since the scMultisim R package lacks the interface to simulate the data resembling real cell type, we implemented scMultisim with the same settings (Li, et al., 2022) as our framework.

For benchmarking clustering methods, we tested Leiden clustering, k-means clustering and Hierarchical clustering (HC) methods (Chen, et al., 2019) using the simulated data generated by simCAS in the discrete mode. For benchmarking trajectory inference methods, we tested Monocle3 (Cao, et al., 2019) with different parameters. For benchmarking methods of data integration or cis-regulatory interaction inference, we also tested Harmony (Korsunsky, et al., 2019) and Cicero (Pliner, et al., 2018) on synthetic data with multiple batches and cis-regulatory interactions, respectively.

For data visualization, we first select 50000 highly accessible peaks, then perform term frequency-inverse document frequency (TF-IDF) and principal component analysis (PCA) transformation to reduce dimensions to 50, and finally apply uniform manifold approximation and projection (UMAP) to project the cells into a 2-dimensional space. Unless otherwise stated, we perform the above pipeline for visualization in this study.

Supplementary Note 6: Evaluation metrics

Suppose R is the sorted real statistic values S is the sorted simulated statistic values, MAD, MAE and RMSE are calculated as following equations:

$$MAD(R,S) = median(|R-S|)$$

$$MAE(R,S) = mean(|R-S|)$$

$$RMSE(R,S) = \sqrt{mean((R-S)^2)}$$

PCC calculates linear correlation between R and S:

$$PCC(R,S) = \frac{\mathrm{cov}(R,S)}{\sigma_R \sigma_S},$$

where cov is the variance and σ is the standard deviation.

JSD is a measurement of the similarity between two probability distributions. For the reason that integral of continuous variable is incapable be calculated directly, JSD is merely calculated to compare discrete statistic peak mean:

$$JSD(R,S) = \frac{1}{2}D(R||M) + \frac{1}{2}D(S||M)$$

$$M = \frac{1}{2}(R+S)$$

$$D(P||Q) = \Sigma_x P(x)\log\left(\frac{P(x)}{Q(x)}\right),$$

where D is Kullback–Leibler divergence, P and Q are two probability distributions.

KS statistic quantifies the distance between the empirical distribution of two samples, which is derived from a nonparametric test of the equality of two continuous samples named KS test. We apply it on continuous statistics such as the log-transformed library size and the cell sparsity.

Median integration local inverse Simpson's index (miLISI) is a score first proposed to evaluate the integration of data with different batches. Gaussian kernel-based distributions of neighborhoods of the mixing batches are built in low-dimensional embedding space. iLISI is then computed for each neighborhood:

$$\mathrm{iLISI} = \frac{1}{\Sigma_{b=1}^{B} p(b)},$$

where $p(b)$ refers to the probability that two sampling neighbors are from the same batch b and B is the number of batches. By considering original data and synthetic data as different batches, this score can directly be adapted to quantify the similarity between synthetic cells and real cells. This score ranges

from 1 to 2, and a larger value indicates a greater similarity. The closer miLISI is to 2, the local neighborhood has more equal synthetic and real cells. We calculate miLISI value on the 2D UMAP embedding space containing real and synthetic cells using the R package LISI.

Denoting ground truth with gt and clustering labels with $pred$. Homo is calculated using:

$$Homo(gt, pred) = 1 - \frac{H(gt|pred)}{H(gt)},$$

where H is the entropy and $H(gt|pred)$ is the conditional entropy of ground truth clusters given the unsupervised predictions.

AMI is calculated using:

$$AMI(gt, pred) = \frac{MI - E[MI]}{mean\big(H(gt), H(pred)\big) - E[MI]},$$

where MI is the mutual information and $E(\cdot)$ is the expectation function.

ARI is calculated using:

$$ARI(gt, pred) = \frac{RI - E[RI]}{\max(RI) - E[RI]},$$

where Rand index (RI) is a similarity measurement between ground truth labels and predicted labels.

F1 score is calculated as follows:

$$\mathrm{F1} = \frac{TP}{TP + \frac{1}{2}(FP + FN)},$$

where TP, FP, TN, FN represent true positive, false positive, true negative and false negative, respectively.

Supplementary Note 7: Analysis of the computing resources

To measure the CPU time and memory usage of simCAS, we performed simulations with varying cell numbers (500, 1,000, 2,000, and 5,000) and peak numbers (10,000, 20,000, 50,000, and 100,000), while keeping other parameters at their default values. The measurement interval for CPU time and memory usage encompasses the period from reading the training dataset to the completion of peak-by-cell matrix generation. The peak-by-cell matrix of Buenrostro2018 dataset (Buenrostro, et al., 2018) is used as the training dataset for the comparisons across different simulators. Memory usage is calculated as the peak value recorded during the simulation. For python-based simulators, we employed the tracemalloc Python module to determine memory usage, while the R package peakRAM was utilized for R-based simulators.

Supplementary Fig. S7a-b presents a comprehensive analysis of simCAS's performance based on CPU time and memory usage across three different modes with varying parameter settings of peak number and cell number. Supplementary Fig. S7a demonstrates a consistent linear increase in CPU time with an increasing cell number for each simulation mode. Interestingly, certain isolated scenarios exhibit shorter CPU times with smaller peak numbers. For instance, the CPU time with 50,000 peaks is observed to be shorter than that with 100,000 peaks. It is worth noting that the cell-wise correction within this step tends to be more time-consuming than the peak-wise correction. Consequently, due to random sampling variations, simulations may require less time with a higher peak number while maintaining the same cell number. Supplementary Fig. S7b showcases the linear growth pattern of memory usage, which corresponds to both the cell number and peak number. This can be attributed to the predominant factor occupying storage space in simCAS, namely the generated parameter matrix. Each element of this matrix represents a non-zero floating-point value. Notably, generating a peak-by-cell matrix with 100,000 peaks and 5,000 cells in pseudo-cell-type mode requires approximately 120 seconds and 8 GB of memory, suggesting that simCAS can be compatible with personal laptops. To summarize, considering the linear increase in CPU time and memory usage with both cell number and peak number, simCAS can be served as a valuable simulator for generating scCAS data with a substantial number of cells and peaks.

We further conducted a comparative analysis of simCAS, simATAC and scMultisim, focusing on their CPU time and memory usage. Using the peak-by-cell matrix of Buenrostro2018 dataset as training

data, we first fixed the peak number of simulated data to 169,221 (as the real Buenrostro2018 dataset) and varied the cell number from 500 to 5,000 to examine the impact of cell number on CPU time and memory usage. Supplementary Fig. S7c illustrates the relationship between cell number and computing resourses required for simCAS, scMultisim, and simATAC. Both simCAS and scMultisim exhibit a linear proportionality with respect to the cell number, indicating increased computing resources as the number of simulated cells grows. Conversely, simATAC displays a less pronounced sensitivity to changes in the number of simulated cells. When simulating a large number of cells, scMultisim exhibits the longest computation time, while simCAS requires the largest memory allocation. We then fixed the cell number of simulated data to 2,000 and varied the peak number from 10,000 to 100,000. As shown in Supplementary Fig. S7d, the memory usage of simCAS is linearly proportional to the peak number, while the CPU time is not that sensitive to the increments of the peak number. For scMultisim, both the CPU time and memory usage demonstrate a linear correlation with the peak number. In contrast, simATAC displays relatively stable computing resource utilization across different peak number settings. The results align with the underlying principles of each simulator. In the framework of simCAS, parameter matrix correction step consumes the most time and occupies the most storage space, and cell-wise correction is significantly more time-cosuming than the peak-wise correction. In the framework of scMultisim, the key step that significantly impacts computing resources is the value sorting of the parameter matrix, with CPU time and memory usage governed by both peak number and cell number. Conversely, simATAC primarily consumes computing resources during the estimation of the input peak-by-cell matrix, suggesting that the CPU time and memory usage of simATAC are primarily influenced by the shape of the input peak-by-cell matrix rather than the simulation matrix itself.

Supplementary Figures

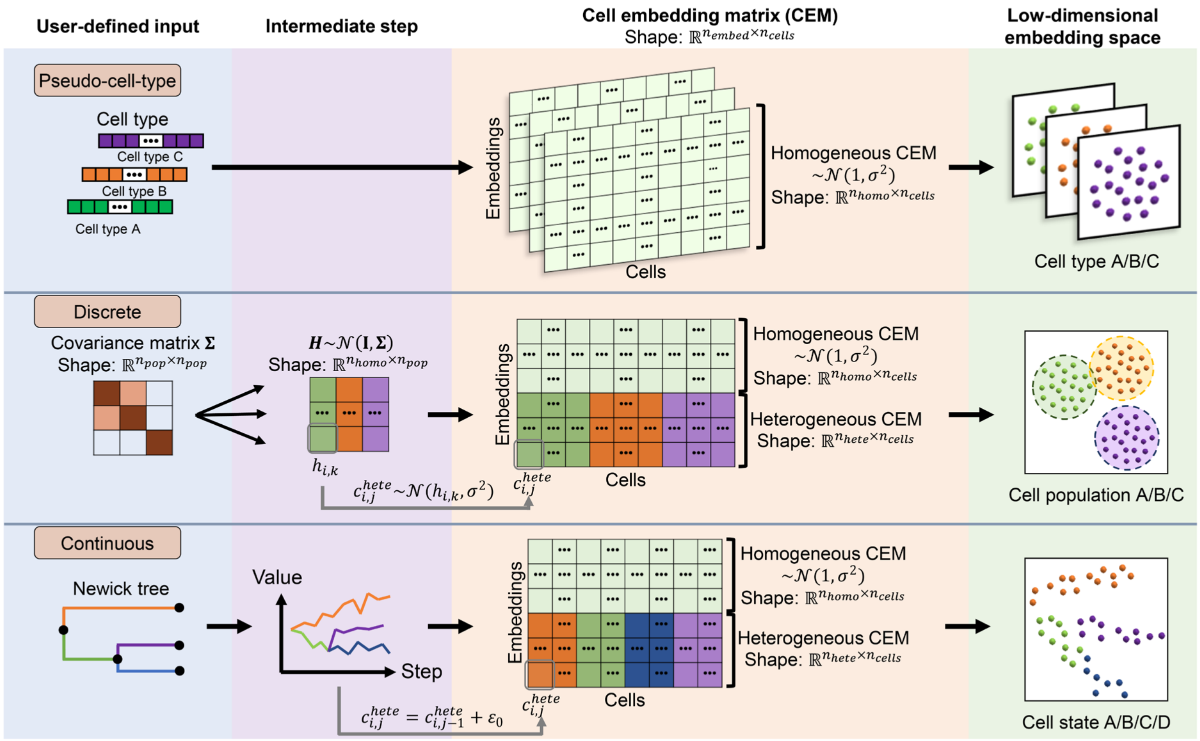

Supplementary Fig. S1. The illustration of details of cell embedding matrix (CEM) generation for different modes. For pseudo-cell-type mode, the cell embeddings are generated from the same Gaussian distribution for different cell types, and the real manifold can be captured by the parameter matrix correction step. For discrete mode, the cell embeddings are generated based on the input covariance matrix, the values of which encode the relationship of cell populations. For continuous mode, the cell embeddings are generated from an input Newick tree, which encodes the predefined differentiation trajectories.

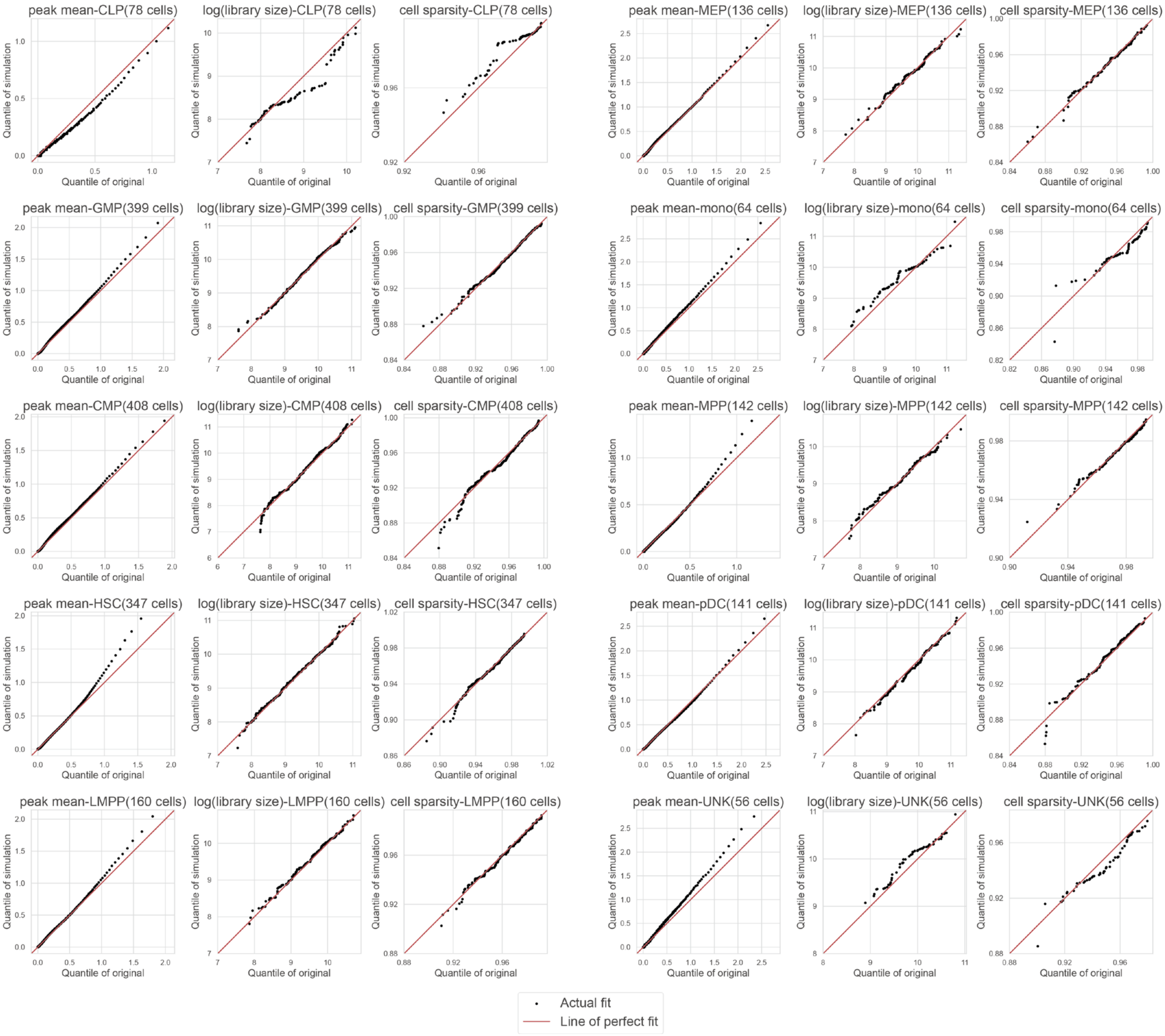

Supplementary Fig. S2. The comparison of the distributions of peak mean, library size and cell sparsity between real data and synthetic data for 10 cell types in Buenrostro2018 dataset.

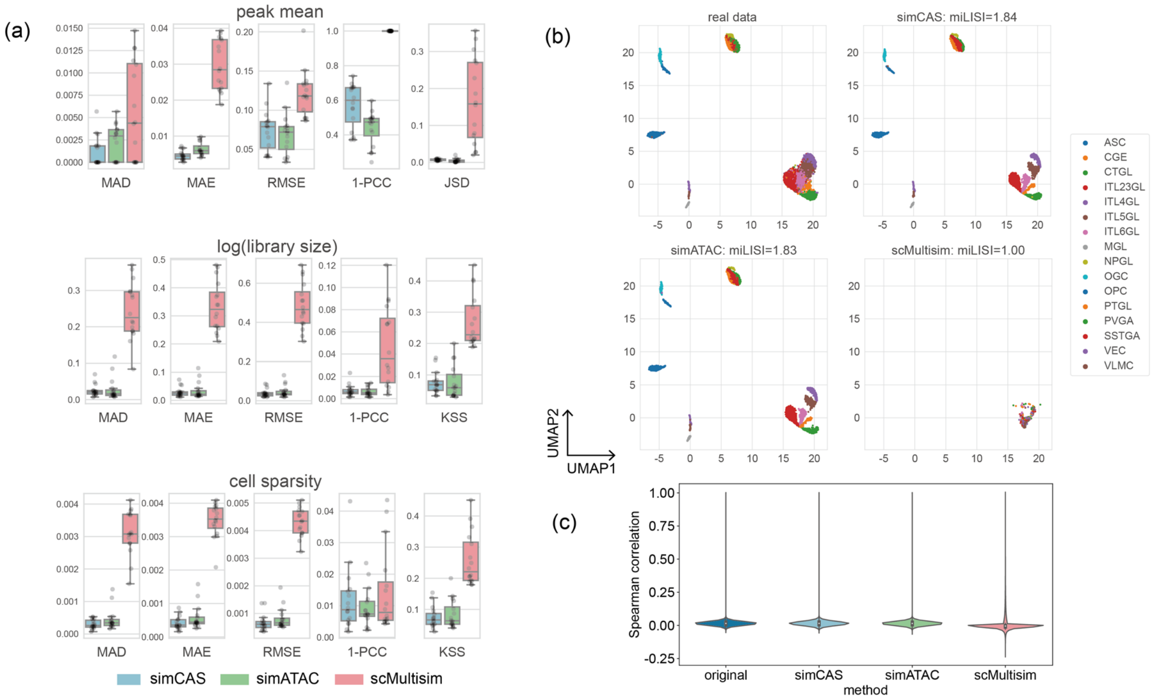

Supplementary Fig. S3. Comparisons of synthetic data and real data in Li2021 dataset. (a) Comparison results of three statistics between synthetic cell types and real cell types measured by six metrics. (b) UMAP visualization of the synthetic datasets and real dataset. (c) Spearman correlation coefficients of synthetic and real datasets on the pairs of top 2000 highly variable peaks selected in real data.

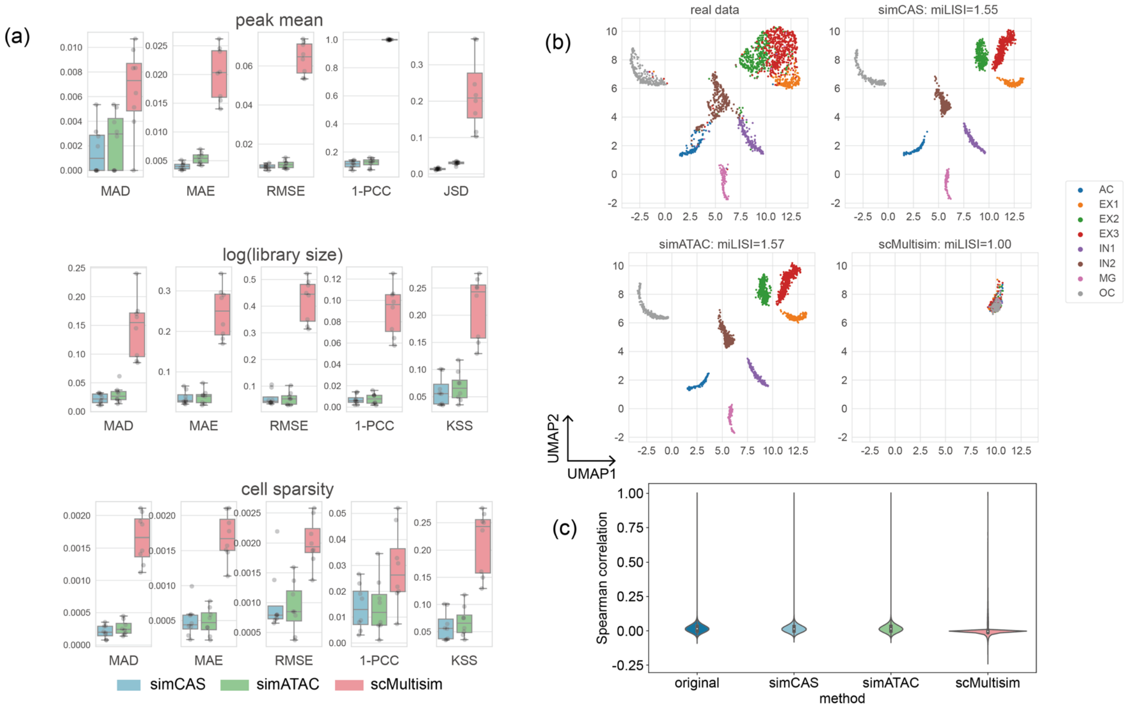

Supplementary Fig. S4. Comparisons of synthetic data and real data in Pressl2018 dataset. (a) Comparison results of three statistics between synthetic cell types and real cell types measured by six metrics. (b) UMAP visualization of the synthetic datasets and real dataset. (c) Spearman correlation coefficients of synthetic and real datasets on the pairs of top 2000 highly variable peaks selected in real data.

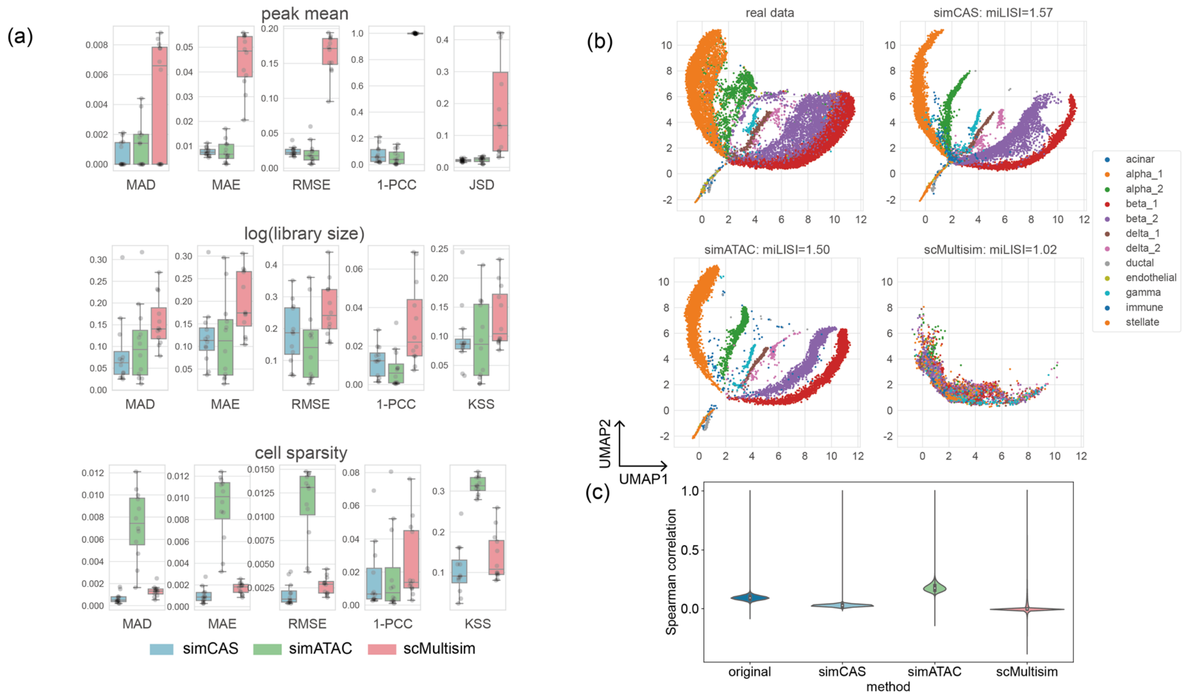

Supplementary Fig. S5. Comparisons of synthetic data and real data in Chiou2021 dataset. (a) Comparison results of three statistics between synthetic cell types and real cell types measured by six metrics. (b) UMAP visualization of the synthetic datasets and real dataset. (c) Spearman correlation coefficients of synthetic and real datasets on the pairs of top 2000 highly variable peaks selected in real data.

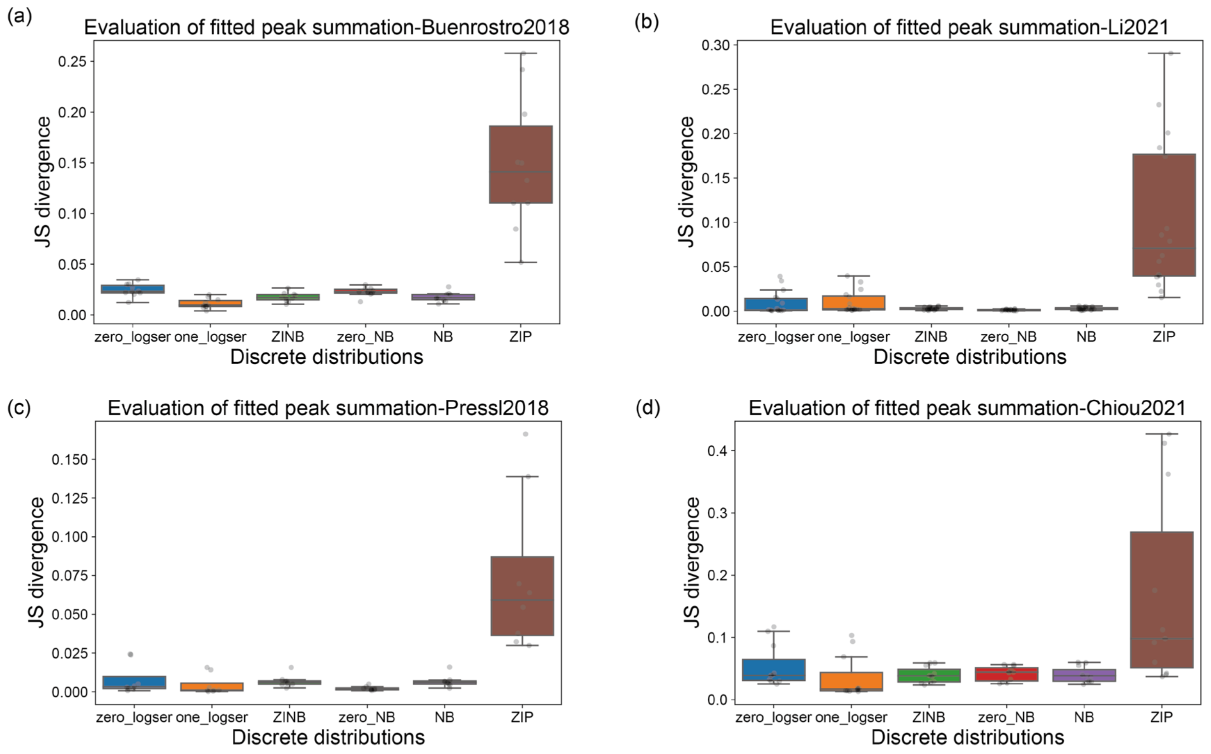

Supplementary Fig. S6. Estimation performance of six discrete distributions on fitting the peak summation of real datasets. The estimation performance is measured by JS divergence. Four real datasets are utilized in the evaluation: (a) Buenrostro2018 dataset, (b) Li2021 dataset, (c) Pressl2018 dataset, and (d) Chiou2021 dataset.

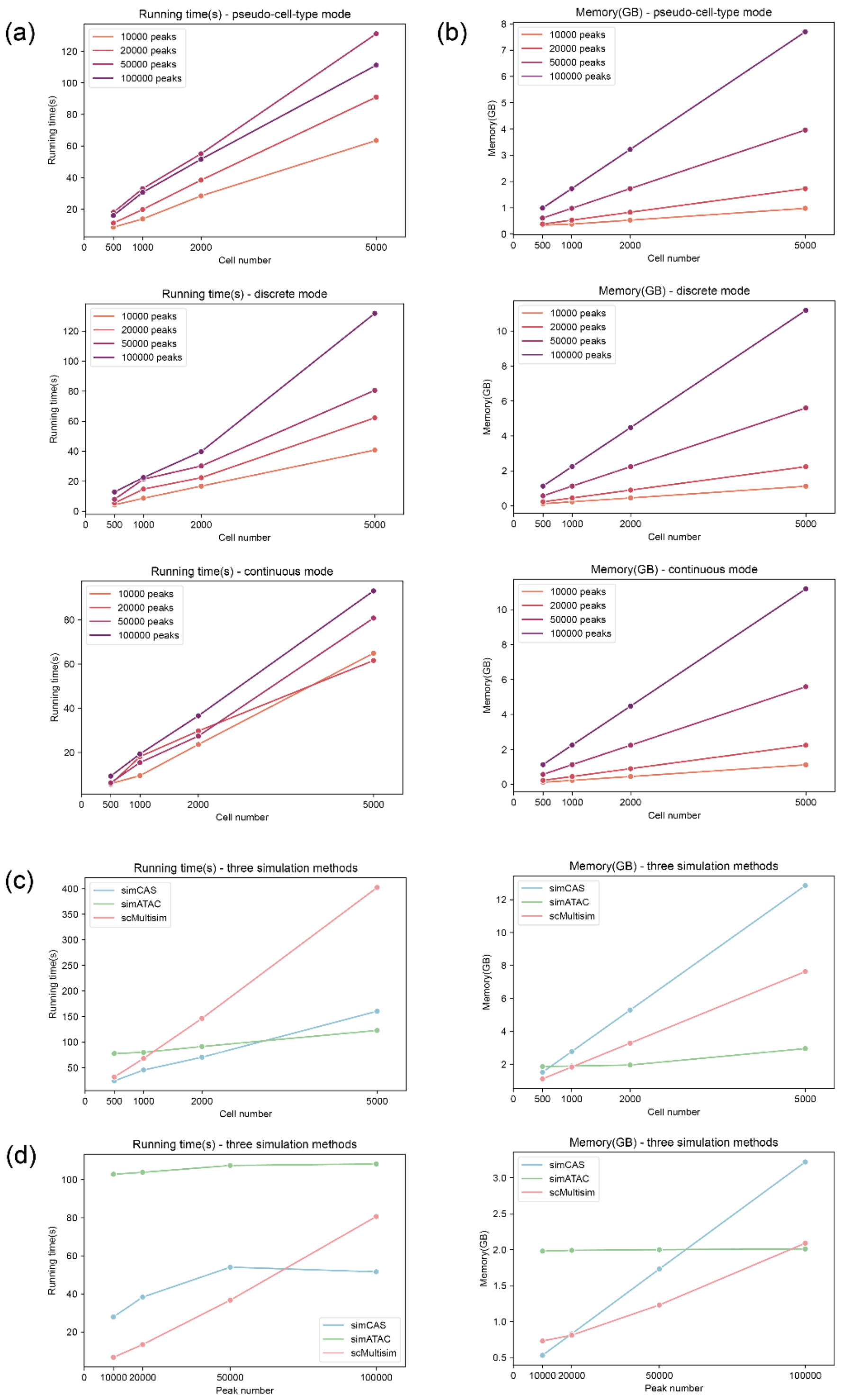

Supplementary Fig. S7. The analysis for computing resources. (a-b) The running time (a) and memory usage (b) of simCAS across three modes with varying parameter settings of peak number and cell number. (c-d) The CPU time and memory usage of simCAS, simATAC, and scMultisim with varying parameter settings of peak number (c) and cell number (d).

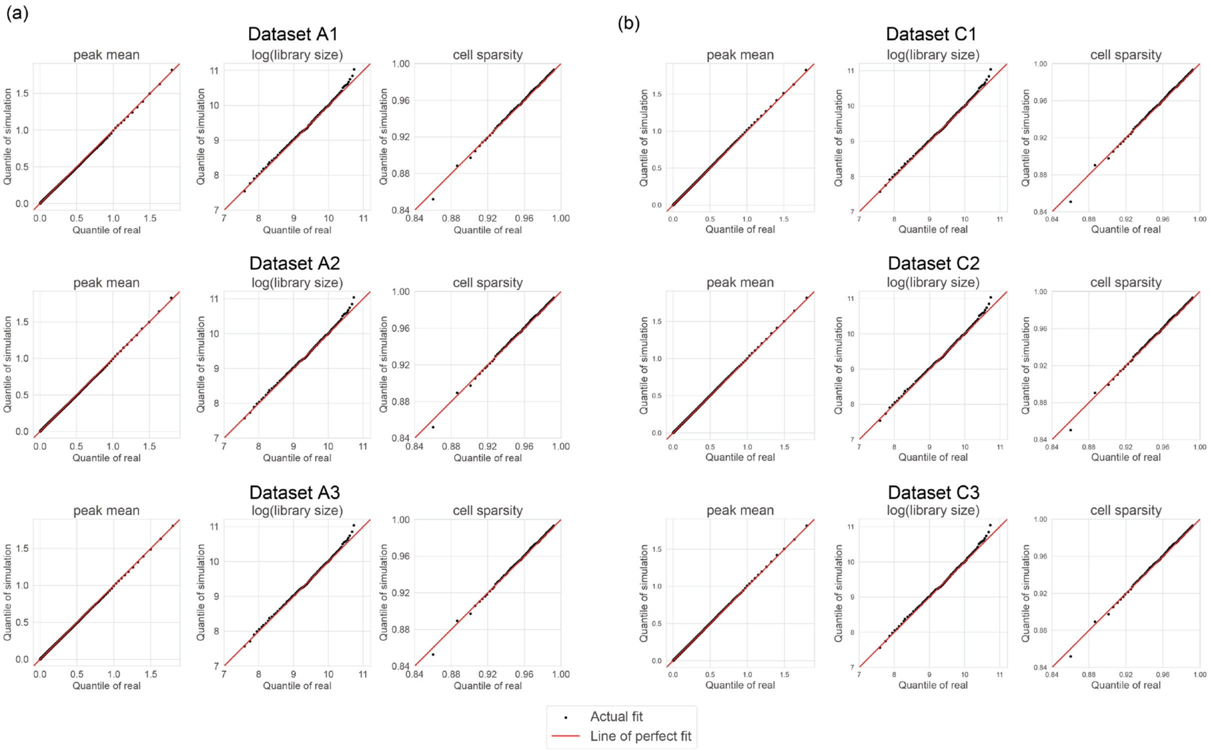

Supplementary Fig. S8. QQ-plots of statistics' comparison between real data and synthetic data. (a) Comparison for datasets A1-A3. (b) Comparison for datasets C1-C3.

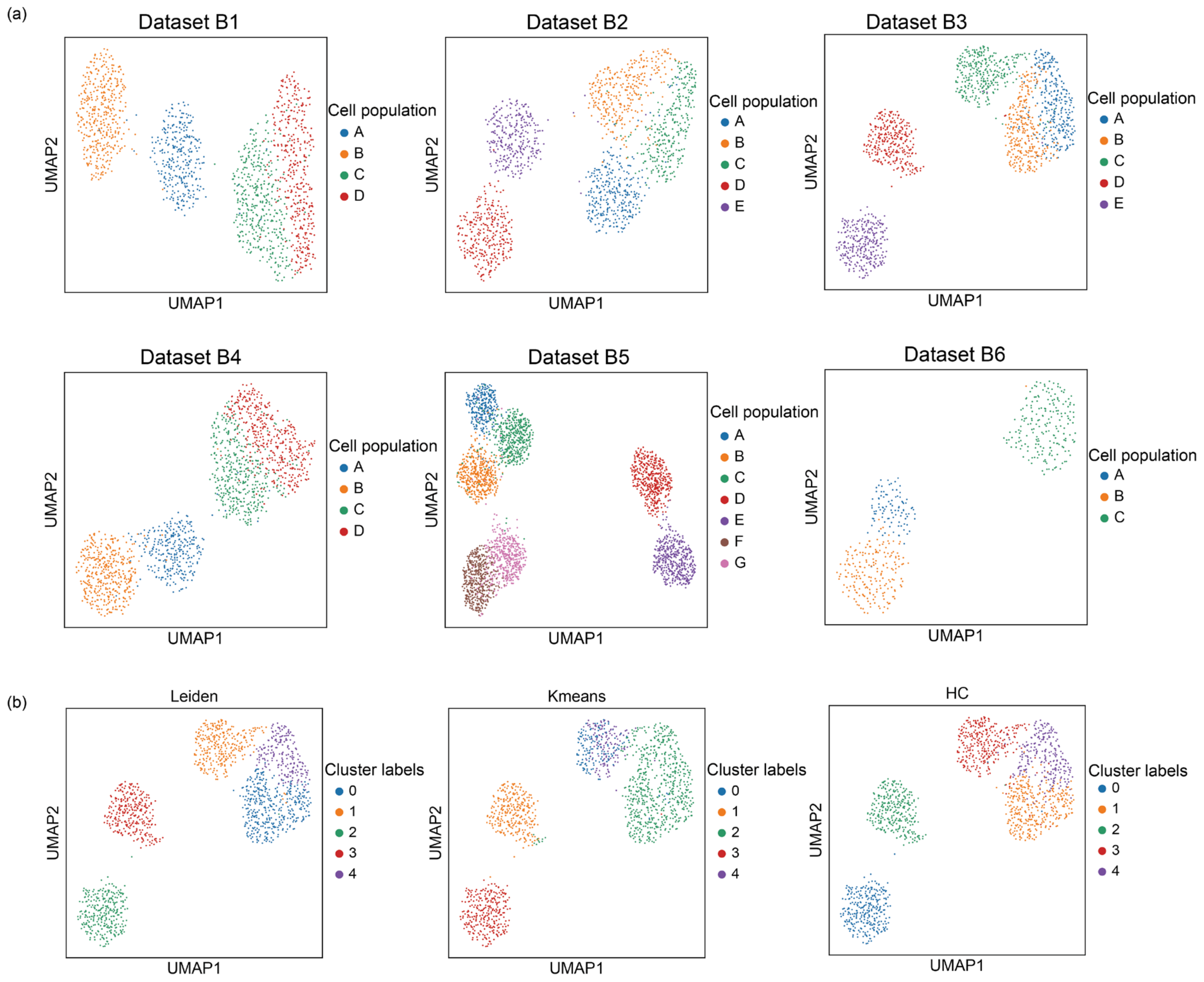

Supplementary Fig. S9. UMAP visualization of the simulated datasets for cell clustering benchmarking and the clustering result. (a) UMAP visualization of datasets B1-B6, colored by the predefined cell populations. (b) An illustration of the clustering result of Leiden clustering, K-means clustering and hierarchical clustering applied on the dataset B3.

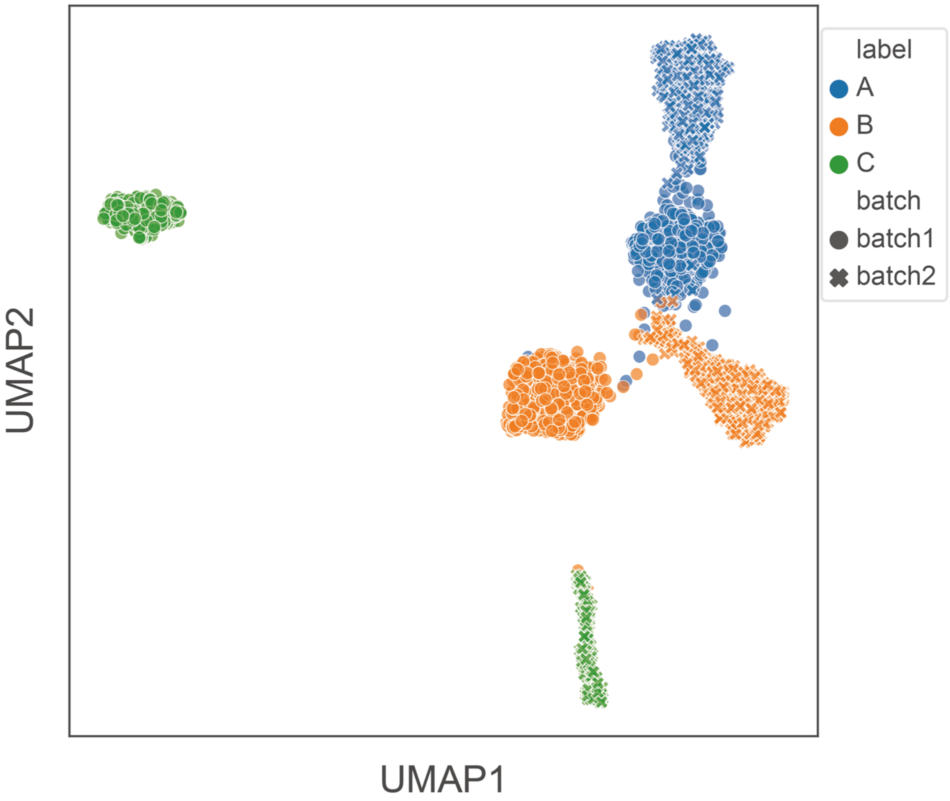

Supplementary Fig. S10. UMAP visualization of a synthetic dataset with simulated technical batch effect. Data of batch1 is generated with populations of 3 (the number of cells in cell population A, B and C is set to 600, 600 and 300, respectively) and a unit diagonal matrix as the covariance matrix. Denote $\boldsymbol{\Lambda}_i$ is part of $\boldsymbol{\Lambda}$ specifically for ith cell population, add Gaussian noise on $\boldsymbol{\Lambda}_i$ proportional to the average cell-wise summations of $\boldsymbol{\Lambda}_i$ to generate data batch2 with technical batch effect compared to data batch1. In this experiment the Gaussian noise is added with mean of 1.88, 0.66 and 0.50 for population A, B and C, respectively, and a fixed standard deviation of 0.5. Then the matrices of data batch1 and data batch2 are concatenated to acquire the data with technical batch effect.

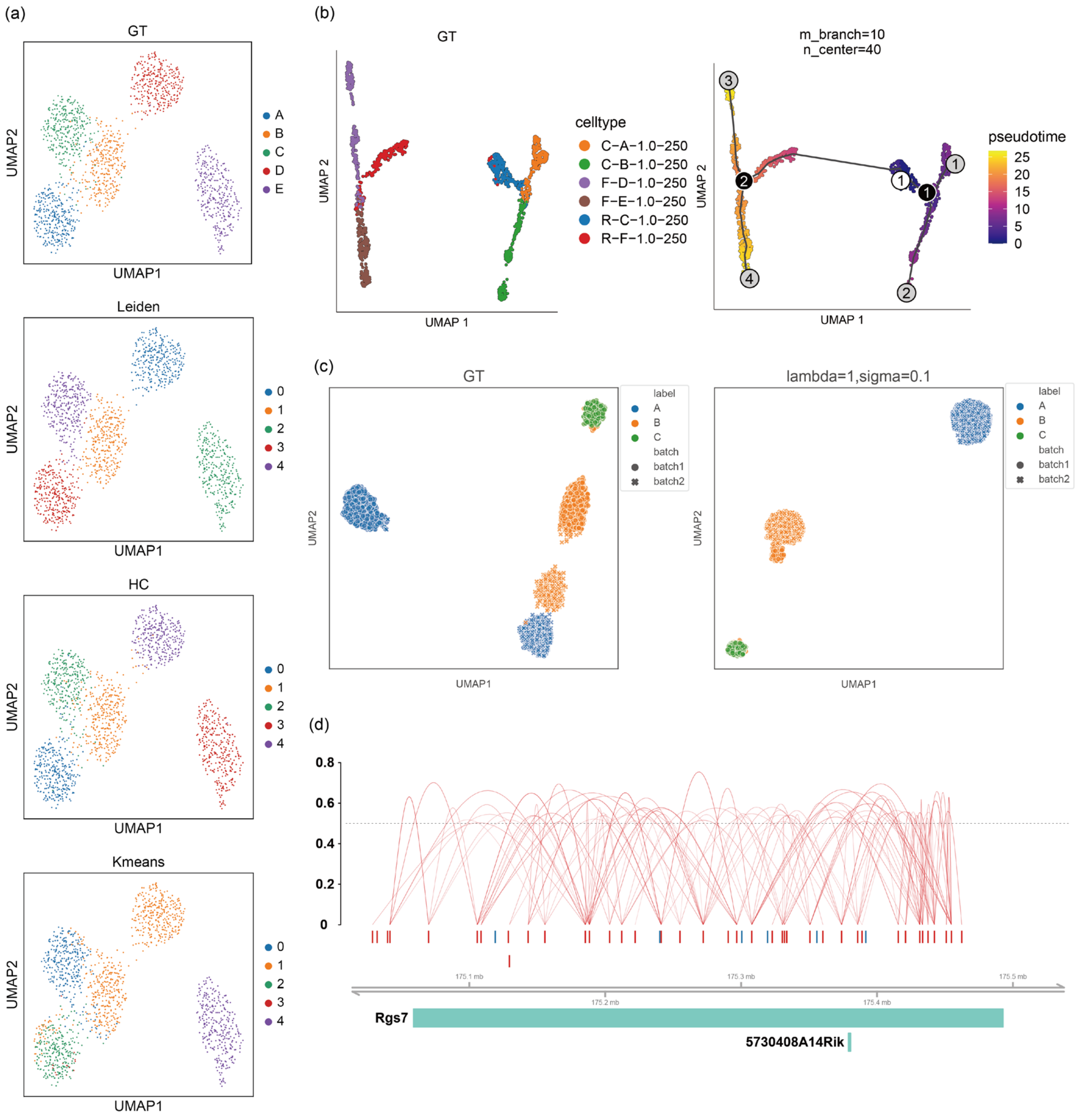

Supplementary Fig. S11. The illustration of four benchmark tasks on the synthetic data generated by simCAS_Bernoulli using Pressl2018 dataset as input. Except for the difference described in Supplementary Note 1, the generation of these binary synthetic matrices is consistent with the descriptions in the manuscript. (a) UMAP visualization of the clustering results of Leiden clustering, K-means clustering and Hierarchical clustering on the simulated discrete data. (b) UMAP visualization of cells with ground-truth trajectories and the inference result of Monocle3. (c) UMAP visualization of cells with technical batch effects and the integration result of Harmony. (d) The predicted connections by Cicero on the peak hub of gene *Rgs7* region.

Supplementary Table S1 Summary of datasets used in this study.

Dataset	Tissue	Protocol	Number of cells	Number of peaks	Number of cell types	Data accession
Buenrostro2018	Blood (human)	scATAC-seq (Fluidigm C1)	1,931	169,221	10	GSE96772
Li2021	Cotex (mouse)	snATAC-seq	5,532	154,308	16	GSM5273008
Pressl2018	Forebrain (mouse)	snATAC-seq	2,062	115,763	10	GSE100033
Chiou2021	Islet (human)	snATAC-seq	15,298	91,754	12	GSE160472

Supplementary Table S2. Comparisons of statistics between synthetic data and real data by metrics.

	Buenrostro2018														
	MAD			MAE			RMSE			1-PCC			KSS/JSD		
	simCAS	simATAC	scMultiSim	simCAS	simATAC	scMultiSim	simCAS	simATAC	scMultiSim	simCAS	simATAC	scMultiSim	simCAS	simATAC	scMultiSim
Peak mean	**0.005±0.003**	0.011±0.005	0.032±0.014	**0.023±0.008**	0.030±0.011	0.178±0.044	**0.063±0.022**	0.071±0.026	0.478±0.094	**0.032±0.020**	0.042±0.021	0.999±0.002	**0.013±0.004**	0.025±0.008	0.119±0.056
log(library szie)	**0.075±0.056**	0.094±0.043	0.151±0.068	**0.088±0.060**	0.105±0.040	0.179±0.077	**0.112±0.073**	0.127±0.043	0.235±0.087	**0.009±0.010**	0.010±0.008	0.023±0.018	**0.091±0.055**	0.112±0.036	0.154±0.043
Cell sparsity	**0.002±0.002**	0.032±0.013	0.005±0.002	**0.003±0.002**	0.032±0.009	0.005±0.002	**0.004±0.002**	0.035±0.009	0.007±0.003	**0.014±0.016**	0.017±0.011	0.016±0.006	**0.094±0.058**	0.433±0.070	0.159±0.044
Peak variance	**0.017±0.011**	0.020±0.009	0.063±0.027	0.303±0.092	**0.161±0.040**	0.406±0.111	2.717±0.913	**0.676±0.217**	1.288±0.406	0.226±0.060	**0.163±0.049**	0.999±0.002	0.194±0.036	**0.177±0.029**	0.421±0.116
Cell variance	0.209±0.100	0.109±0.035	**0.045±0.027**	0.240±0.088	0.152±0.040	**0.060±0.038**	0.299±0.108	0.231±0.112	**0.109±0.078**	**0.023±0.022**	0.039±0.047	0.024±0.014	0.320±0.067	0.330±0.085	**0.159±0.051**
Peak sparsity	**0.003±0.003**	0.012±0.005	0.015±0.006	**0.017±0.005**	0.035±0.009	0.068±0.016	**0.042±0.009**	0.068±0.013	0.149±0.024	0.162±0.025	**0.037±0.018**	0.999±0.002	0.028±0.004	**0.021±0.005**	0.126±0.057
	Li2021														
	MAD			MAE			RMSE			1-PCC			KSS		
	simCAS	simATAC	scMultiSim	simCAS	simATAC	scMultiSim	simCAS	simATAC	scMultiSim	simCAS	simATAC	scMultiSim	simCAS	simATAC	scMultiSim
Peak mean	**0.001±0.002**	0.002±0.002	0.006±0.005	**0.005±0.001**	0.006±0.002	0.030±0.007	0.074±0.025	**0.069±0.026**	0.121±0.028	0.571±0.123	**0.439±0.096**	1.000±0.001	0.007±0.002	**0.004±0.005**	0.166±0.113
log(library szie)	**0.025±0.015**	0.029±0.030	0.243±0.076	**0.030±0.017**	0.035±0.028	0.332±0.086	**0.040±0.020**	0.047±0.030	0.484±0.114	0.007±0.005	**0.006±0.004**	0.047±0.035	**0.075±0.034**	0.077±0.051	0.271±0.082
Cell sparsity	**0.000±0.000**	0.000±0.000	0.003±0.001	**0.000±0.000**	0.001±0.000	0.003±0.000	**0.001±0.000**	0.001±0.000	0.004±0.001	0.012±0.010	**0.009±0.006**	0.014±0.013	**0.074±0.033**	0.084±0.044	0.260±0.082
Peak variance	**0.001±0.002**	0.002±0.002	0.006±0.005	**0.007±0.003**	0.009±0.003	0.034±0.012	0.623±0.436	**0.617±0.437**	1.032±0.829	0.865±0.051	**0.691±0.059**	1.000±0.000	0.065±0.019	**0.052±0.037**	0.451±0.213
Cell variance	**0.005±0.003**	0.006±0.004	0.007±0.002	**0.008±0.006**	0.008±0.007	0.011±0.005	**0.014±0.012**	0.014±0.013	0.025±0.014	0.091±0.140	**0.079±0.126**	0.090±0.075	**0.319±0.097**	0.335±0.103	0.357±0.070
Peak sparsity	**0.001±0.002**	0.002±0.002	0.006±0.005	**0.004±0.001**	0.006±0.002	0.028±0.006	**0.009±0.003**	0.012±0.004	0.079±0.011	**0.056±0.026**	0.075±0.042	1.000±0.003	0.007±0.002	**0.004±0.005**	0.167±0.113
	Pressl2018														
	MAD			MAE			RMSE			1-PCC			KSS		
	simCAS	simATAC	scMultiSim	simCAS	simATAC	scMultiSim	simCAS	simATAC	scMultiSim	simCAS	simATAC	scMultiSim	simCAS	simATAC	scMultiSim
Peak mean	**0.002±0.002**	0.003±0.002	0.007±0.003	**0.004±0.001**	0.005±0.001	0.020±0.004	**0.008±0.001**	0.010±0.002	0.064±0.008	**0.111±0.025**	0.123±0.030	1.000±0.003	**0.014±0.002**	0.030±0.004	0.224±0.096
log(library szie)	**0.022±0.008**	0.030±0.014	0.146±0.050	**0.036±0.015**	0.039±0.016	0.247±0.060	**0.056±0.026**	0.054±0.024	0.421±0.076	**0.007±0.004**	0.008±0.005	0.091±0.022	**0.061±0.025**	0.069±0.026	0.215±0.055
Cell sparsity	**0.000±0.000**	0.000±0.000	0.002±0.000	**0.000±0.000**	0.000±0.000	0.002±0.000	**0.001±0.000**	0.001±0.000	0.002±0.000	**0.014±0.008**	0.014±0.010	0.029±0.014	**0.061±0.025**	0.069±0.026	0.215±0.055
Peak variance	**0.002±0.002**	0.002±0.002	0.007±0.003	**0.004±0.001**	0.005±0.001	0.016±0.003	**0.007±0.001**	0.009±0.002	0.036±0.003	**0.095±0.020**	0.129±0.031	1.000±0.003	**0.111±0.014**	0.172±0.023	0.560±0.147
Cell variance	**0.000±0.000**	0.000±0.000	0.002±0.000	**0.000±0.000**	0.000±0.000	0.002±0.000	**0.001±0.000**	0.001±0.000	0.002±0.000	**0.013±0.008**	0.014±0.010	0.029±0.014	**0.061±0.025**	0.069±0.026	0.215±0.054
Peak sparsity	**0.002±0.002**	0.003±0.002	0.007±0.003	**0.004±0.001**	0.005±0.001	0.020±0.004	**0.008±0.001**	0.010±0.002	0.064±0.008	**0.111±0.025**	0.123±0.030	1.000±0.003	**0.014±0.002**	0.030±0.004	0.224±0.096
	Chiou2021														
	MAD			MAE			RMSE			1-PCC			KSS		
	simCAS	simATAC	scMultiSim	simCAS	simATAC	scMultiSim	simCAS	simATAC	scMultiSim	simCAS	simATAC	scMultiSim	simCAS	simATAC	scMultiSim
Peak mean	**0.001±0.001**	0.001±0.001	0.004±0.004	**0.008±0.002**	0.008±0.004	0.045±0.011	0.024±0.006	**0.022±0.015**	0.163±0.027	0.078±0.062	**0.057±0.052**	0.999±0.003	**0.022±0.005**	0.024±0.009	0.183±0.150
log(library szie)	**0.086±0.078**	0.105±0.085	0.158±0.056	0.122±0.068	**0.118±0.089**	0.196±0.067	0.186±0.091	**0.148±0.109**	0.261±0.084	0.012±0.008	**0.008±0.009**	0.031±0.021	0.097±0.054	**0.094±0.065**	0.133±0.050
Cell sparsity	**0.001±0.000**	0.007±0.003	0.001±0.000	**0.001±0.001**	0.009±0.003	0.002±0.001	**0.002±0.001**	0.011±0.004	0.003±0.001	**0.016±0.020**	0.019±0.025	0.025±0.023	**0.106±0.059**	0.314±0.021	0.139±0.057
Peak variance	**0.003±0.002**	0.003±0.003	0.009±0.008	0.053±0.016	**0.029±0.005**	0.083±0.020	0.420±0.126	**0.115±0.045**	0.276±0.050	0.229±0.119	**0.153±0.134**	0.999±0.003	**0.171±0.050**	0.194±0.051	0.451±0.241
Cell variance	0.033±0.018	0.019±0.007	**0.007±0.002**	0.040±0.020	**0.025±0.008**	0.009±0.003	0.055±0.026	**0.035±0.009**	0.018±0.008	0.032±0.037	**0.029±0.025**	0.030±0.026	**0.267±0.057**	0.323±0.069	0.130±0.052
Peak sparsity	**0.001±0.001**	0.002±0.002	0.002±0.002	**0.007±0.002**	0.011±0.003	0.021±0.005	**0.019±0.003**	0.028±0.007	0.067±0.011	**0.249±0.030**	0.054±0.048	0.999±0.003	**0.033±0.013**	0.036±0.010	0.184±0.150

References

Cao, J., *et al.* The single-cell transcriptional landscape of mammalian organogenesis. *Nature* 2019;566(7745):496-502.

Chen, H., *et al.* Assessment of computational methods for the analysis of single-cell ATAC-seq data. *Genome Biol* 2019;20(1):241.

Korsunsky, I., *et al.* Fast, sensitive and accurate integration of single-cell data with Harmony. *Nat Methods* 2019;16(12):1289-1296.

Li, H., *et al.* scMultiSim: simulation of multi-modality single cell data guided by cell-cell interactions and gene regulatory networks. *bioRxiv* 2022.

Navidi, Z., Zhang, L. and Wang, B. simATAC: a single-cell ATAC-seq simulation framework. *Genome Biol* 2021;22(1):74.

Pliner, H.A., *et al.* Cicero Predicts cis-Regulatory DNA Interactions from Single-Cell Chromatin Accessibility Data. *Mol Cell* 2018;71(5):858-871 e858.
